# Improving the sexual activity and reproduction of female zebrafish with high testosterone levels

**DOI:** 10.1038/s41598-021-83085-4

**Published:** 2021-02-15

**Authors:** Congcong Liu, Sophie Yue, Joshua Solarz, Jessica Lee, Lei Li

**Affiliations:** 1 Center for Reproductive Medicine, Shandong University, Jinan, 250012 China; 2 Department of Biological Sciences, University of Notre Dame, Notre Dame, IN 46556 USA

**Keywords:** Neuroscience, Diseases

## Abstract

High levels of testosterone cause clinical symptoms in female reproduction and possibly, alterations in sexuality. Yet, the underlying mechanisms remain to be examined. Here, we report a study that investigates the effects of testosterone in follicle development and sexual mating using zebrafish models. We developed an acute zebrafish model with high testosterone levels by exposing young female zebrafish to testosterone dissolved in swimming water. After given a high concentration of testosterone treatment (e.g., 100 ng/ml), the fish showed hallmark pathological symptoms similar to those displayed in patients with polycystic ovary syndrome (PCOS), such as follicular growth-arrest, rare ovulation, ovary enlargement, decrease in reproduction, and down regulation of the expression of some PCOS susceptible genes, such as *Tox3*. These fish are referred to as the PCOS fish. By monitoring mating-like swimming behaviors, we measured the sexual activity of PCOS zebrafish. In general, the PCOS fish showed no desire to interact with males. As a consequence, their mating rate was decreased as compared to control animals. The sexuality levels of PCOS fish, however, could be improved after short periods of rearing in conditions that lack of males. After only 3 days of rearing alone, the PCOS fish showed an increase in sexuality levels and displayed characteristic swimming patterns for mating. After 30 days of separation from males, not only the sexual activity, but also the mating rate was improved in the PCOS fish. Together, the data suggests that zebrafish can serve as a new type of research model to further develop strategies for the treatment of reproductive disorders, such as those related to PCOS.

## Introduction

In humans, high levels of testosterone lead to active sexual behaviors in males but cause reproductive disorders in females, namely, polycystic ovary syndrome (PCOS)^1–3^. PCOS is characterized by hyperandrogenism, pathologic ovary, oligo-ovulation, which together, lead to clinical consequences such as infertility^4,5^. Previous studies have demonstrated that both genetic and epigenetic factors are attributed to the development of PCOS^6–9^. While gene mutations that cause PCOS have not been identified, a number of genetic loci that are susceptible to PCOS have been discovered^10^. Some of them, such as *Tox3* and *Dennd1a*, are also involved in nervous system development and cell proliferation. In mouse cortical neurons, for example, the expression of *Tox3* is required for calcium-dependent signaling transduction, which in turn, promotes synaptic formation and neural transmission^11^. In cultured human cancer cell lines, through intracellular epidermal growth factor pathways, the expression of *Dennd1a* facilitates cell migration and aggression^12^. Interruptions of endocrine pathways, particularly those involved in hormone synthesis and circulation, may also lead to increases in testosterone levels and the development of PCOS^13–17^. In cultured mouse ovary granulosa cells, for example, accumulation of non-histone chromosomal proteins due to endocrine system malfunctions, which is commonly seen in human patients with high levels of testosterone, inhibits granulosa cell proliferation, which in turn, causes follicular growth-arrest and PCOS^18^.

Treatments for high levels of testosterone in females are widely available, which include hormone adjustment (e.g., to lower the level of androgen and restore menstrual regularity) and ovulation therapy (e.g., to stimulate follicle maturation)^19–25^. They are effective in solving the occurrence of short-term complications, but can sometimes cause serious side effects, such as ovarian hyperstimulation, hyperkalemia, or liver damage^26^.

Animal models, which include mice, rats, and sheep, have been previously described for studying human reproductive disorders caused by high levels of testosterone^27–29^. Recently, zebrafish have emerged as an alternative model organism for studying human diseases^30–33^. Zebrafish are vertebrate organisms with approximately 70% genetic homology to humans^34^. They have a relatively short sexual maturation time (2 months), and a rapid recovery period of ovulation (1–2 week). A young female can lay several hundreds of eggs every other week^35,36^. The eggs are fertilized in water and the embryos are transparent and develop externally, making them an excellent model for studying developmental biology. Zebrafish have a small body size (e.g., adult zebrafish is approximately 1-inch long) and move slowly (e.g., 1–3 cm per second), and their behaviors can be readily observed^37^. Here, we report a study of follicle development and sexual mating in female zebrafish with a high level of testosterone. We proposed that high levels of testosterone interrupt the pathways of signal transduction involved in ovulation and sexuality, which in turn, decreases reproduction. We developed acute zebrafish models with high levels of testosterone, referred to as PCOS fish, and characterized the zebrafish models using molecular, histologic, and behavioral assays. In addition, we developed methods for improving the mating rate and reproductivity in PCOS fish. Together, the data suggests that zebrafish can be used for studying the mechanisms of human diseases involved in female reproduction.

## Materials and methods

### Animal care

Zebrafish colonies (AB strain, between 3 and 8 months of age) were maintained in the fish facility in accordance with the animal protocol approved by the University of Notre Dame IACUC (Protocol #, 17-11-4243). The fish were maintained in 5-L tanks (40 fish per tank, males and females were intermixed) with circulating water (distilled water with instant ocean added, 3 mg/l; pH = 7.0 ± 0.2), in which approximately 10% of the total volume was renewed each day. The male and female fish were identified based on their sexual dimorphisms (e.g., body shape, skin color). The fish facility was set at 28 ± 0.5 °C (room temperature) and illuminated with fluorescent ceiling light in 14:10 light–dark cycles (light on, 7AM; light off, 9PM). The fish were fed twice a day with freshly hatched brine shrimps. The health of zebrafish and the quality of the water were monitored by certified veterinarians and animal-care personnel on a daily basis. For each set of the experiments involving testosterone treatment, histology, and qRT-PCR, 5–8 animals were used. For each set of the behavioral tests, 8–12 animals were used. The studies reported in this paper were conducted in compliance with the ARRIVE guidelines, and the methods were carried out in accordance with the NIH regulations. Major experimental procedures are demonstrated in Fig. S1.

### Testosterone treatment

Testosterone (Biorular, Danbury, USA) was dissolved in distilled water (2.0 × 10^4^ ng/ml) that contained 0.1% dimethyl sulfoxide (DMSO) and stored at − 20 °C. DMSO was used to dissolve testosterone^38^. Prior to each treatment, desired amounts of testosterone stock solutions were added to containers that contained 1000 ml fish water to make the final testosterone concentration at 0.25, 2, 10, or 100 ng/ml. Female zebrafish (20 fish per tank) were allowed to swim in the container under normal rearing conditions (14:10 light–dark cycle, 28 °C, normal feeding) for 3 consecutive days, and the drug solutions were changed once a day. During the treatment, no signs of stress and/or discomfort were observed in individual animals (e.g., jerky movement or remaining still at the bottom of the container). Control treatment was conducted in 0.1% DMSO dissolved in regular facility water. Unless specified, the experiments were conducted using zebrafish that were treated with 100 ng/ml testosterone because this dose of treatment caused significant changes (e.g., gene expression, ovary histology, and sexual activity) as compared to control animals.

After testosterone treatment, the fish were either kept alone or intermixed with males for 3, 14, 30, or 60 days before they were transferred to the mating cage that contained male animals. They were then tested for sexual mating and reproduction.

### Measuring the testosterone levels

Zebrafish were anaesthetized and sacrificed in 1% tricaine mesylate (Sigma-Aldrich, MO, USA) dissolved in water at 4 °C in accordance with the approved animal protocols. Ovary and brain tissues were isolated, weighed, and homogenized using methanol (75% in water; 1 µg tissue in 1 µl solution). Samples were centrifuged at 2000*g* in 4 °C, and the supernatants were collected. These procedures were repeated 3 times, and the supernatants were combined and lyophilized with vacuum and stored in − 80 °C. Testosterone levels were measured using the gas chromatography-mass spectrometry (GCT Premier Mass Spectrometer, coupled with an Agilent 6890 Gas Chromatograph fitted with 7683B Series Injector) in accordance with the manufacturer’s instruction (Waters Corporation, Milford, USA).

### Histology and classification of follicles

Zebrafish was anaesthetized and sacrificed in accordance with the approved animal protocols. Ovaries were dissected and fixed in 4% paraformaldehyde overnight at 4 °C. After dehydration in 80%, 90%, 95%, 100% ethanol and 100% xylene, specimens were embedded in paraffin and frozen at − 20 °C. Sections of 6 μm thickness were collected onto glass slides, air dried, stained with hematoxylin and eosin, and shielded with cover glass. In each ovary, 8 sections at 6 μm intervals were collected.

The sections were examined under a microscope. Based on the size of follicles and the distribution of endocrine contents in surrounding tissues, the follicles were assigned into five groups, which represent the developing, transitioning, and mature follicles^39^. Stage I: primary growth stage, follicle diameter < 140 μm, surrounded by a flat layer of pre-follicle cells; Stage II: cortical alveolus stage, follicle diameter 140–340 μm, basophilic vesicles presented in the cytoplasm of the oocyte; Stage III: vitello genesis stage, follicle diameter 340–690 μm, eosinophilic vesicles presented in cytoplasm of the oocyte; Stage IV: oocyte maturation stage, follicle diameter 690–730 μm, yolk granular cells could be found in cytoplasm of the oocyte; Stage V: mature eggs, diameter > 730 μm, yolk granules could be found in the cytoplasm of the oocyte. From each section, between 30 and 80 follicles were examined depending on the size of the section.

### Reverse transcription and qRT-PCR

RNA was extracted from the isolated ovary and brain using the Trizol reagent kit (Thermo Fisher, Waltham, USA). Using the PrimeScript RT kit with gDNA Eraser (Takara, Dalian, China), 1 μg of total RNA was converted into cDNA. Reactions in 10 μl volume containing 1 μl of cDNA, 5 μl of SYBR green master mix, 1 μl of primer mix, and 3 μl of RNase-free water per sample were conducted using Light Cycler 480 (Roche Applied Science, Basel, Switzerland) with SYBR Premix ExTaq (Takara, Dalian, China) in the following steps: 10 min at 95 °C, followed by 40 cycles of 10 s at 95 °C, 20 s at 60 °C, 10 s at 72 °C. The primer sequences were: Actin: forward: 5′-CAGCCTTCC TTCCTGGGTA-3′, reverse: 5′-TGGCATACAGGTCCTTACGG-3′. *Tox3*: forward: 5′-GTCTGCATA TGCCCTGTTCT-3′, reverse: 5′-TCCCACATGGAGGCTACTAT-3′; *Dennd1a*: forward: 5′-CGCTAT CCAGCTACAGTTCTTC-3′, reverse: 5′-CCATGTTGATCTCCTCCTCAAA-3′; Reaction products were analyzed by comparative C(T) to determine the relative gene expression levels.

### Measuring the gonado-somatic index

The gonado-somatic index (GSI) has been previously used as a criterion for evaluating the development and function of ovaries in animal models^40^. We measured the GSI in both the control and testosterone-induced PCOS zebrafish. The fish was anaesthetized, wiped and dried with a paper towel, and weighed. Then, the fish was sacrificed and the ovaries were dissected and weighed. The GSI was calculated as the following: GSI = ovary weight (g)/body weight (g).

### Videotaping and scoring the sexuality activity

Zebrafish swimming behaviors were videotaped using a camcorder (Sony, CX440) set on a tripod. Recording were made in the early morning, between 07:00 and 07:30 AM, during which time natural mating occurs^36,37^. In the evening before videotaping, the male and female fish (either a control or testosterone-treated PCOS fish) were transferred to the mating cage (20.3 cm width × 7.6 cm depth × 7.6 cm height). Each cage hosted one male fish and one female fish. The mating cage was divided into two sections by an aluminum screen inserted in the middle of the cage. The screen permitted water flow but separated the male and female fish, preventing them from making physical contact with each other. Each half of the cage was further partitioned into 3 sections by lines marked on a paper that was placed underneath the cage. The lines could be visualized through the bottom of the transparent cage. This was to indicate the areas related to the net in the middle of the mating cage.

On the day of the experiment, immediately after light on-set in the morning, we videotaped the fish for 30 min. After videotaping, we analyzed the swimming behaviors of the female fish to determine its tendency for mating. Specifically, we quantified the animal’s desire for mating, referred to the “sexuality levels”^41^. Several measures were put in place to indicate the animal’s sexuality levels. First, we monitored the locations where the fish swam at 15-s intervals. If the fish swam in the section that was closest to the net in the middle of the cage (which was close to the male fish that swam in other half of the cage), the fish was given a score of 5 points; if the fish swam in the middle section of its compartment, the fish was given a score of 3 points; if the fish was recorded in the third section which was farthest from the net, the fish was given a score of 0 points. Second, we determined the speed of fish swimming at 15-s intervals. This was done by measuring the distance (in centimeters) the fish swam in a 1-s period. Fast swimming (e.g., > 3.0 cm/s) valued 2 points, normal swimming (between 1.0–3.0 cm/s) earned 1 point, and slow swimming (e.g., < 1.0 cm/s) received 0 points. Third, we counted the number of times the fish attempted to cross the middle line of the cage (e.g., the number of times the fish approached the net). Each time the fish swam against the net (i.e., physical contact), the fish received an additional 5 points. Total scores were summed for individual fish to indicate its sexuality levels. In all cases, the analysis of fish behaviors on the videotape was performed by researchers who possessed no knowledge of the origins and conditions of the fish (i.e., if the fish on the video was a PCOS fish or a control fish).

### Mating

After videotaping the swimming behaviors, the net in the middle of the mating cage was removed and the female and male zebrafish were allowed to mate for 1 h. The number of females that laid eggs, the number of eggs that spawned from each mating, and the number of embryos that hatched at 5 days post-fertilization were counted.

### Statistical analysis

Data was analyzed using the IBM SPSS Statistics 23.0 and assessed by the Kolmogorov–Smirnov test to determine whether the continuous variables were normally distributed. Normally distributed data was analyzed using the Student’s *t* test, and nonparametric data was analyzed using the Mann–Whitney U test. Statistically significant differences were considered when the p value was smaller than 0.05 (two-tailed).

## Results

### Creating female zebrafish with high levels of testosterone

**Figure 1:**
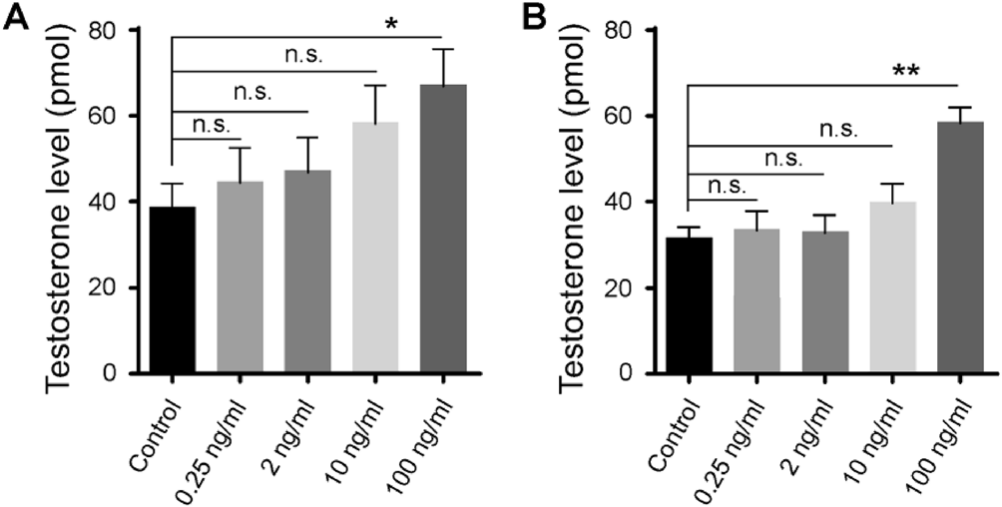
Testosterone levels in isolated ovary (**A**) and brain (**B**) tissues in control (black bars) and drug treated (grey bars) animals. Note the increase of testosterone levels after drug treatment. Data represents the means ± SE (n = 5 in each group). *p < 0.05; **p < 0.01; *ns* not significant.

To create acute female zebrafish models with high levels of testosterone, we treated young adult female fish with different concentrations of testosterone (0, 0.25, 2, 10, and 100 ng/ml) applied to swimming water. After 3 consecutive days of treatment, we isolated their ovary and brain tissues and measured testosterone levels. When treated with a lower concentration of testosterone (e.g., 0 ng/ml and 0.25 ng/ml), no changes in testosterone levels were observed in either organ. With the increase of treatment concentration (e.g., 2 ng/ml and 10 ng/ml), slight increases in testosterone levels were observed, but the differences were not statistically significant as compared to control samples. In zebrafish treated with the highest concentration of drugs (e.g., 100 ng/ml), significant increases of testosterone levels were detected (e.g., 74.5% in the ovary and 86.3% in the brain). Zebrafish that showed significant increases in testosterone levels in the ovaries and in the brain after treatment with 100 ng/ml testosterone were thereby referred to as “PCOS zebrafish” (Fig. 1).

### PCOS zebrafish showed defects in follicle maturation and ovulation

**Figure 2:**
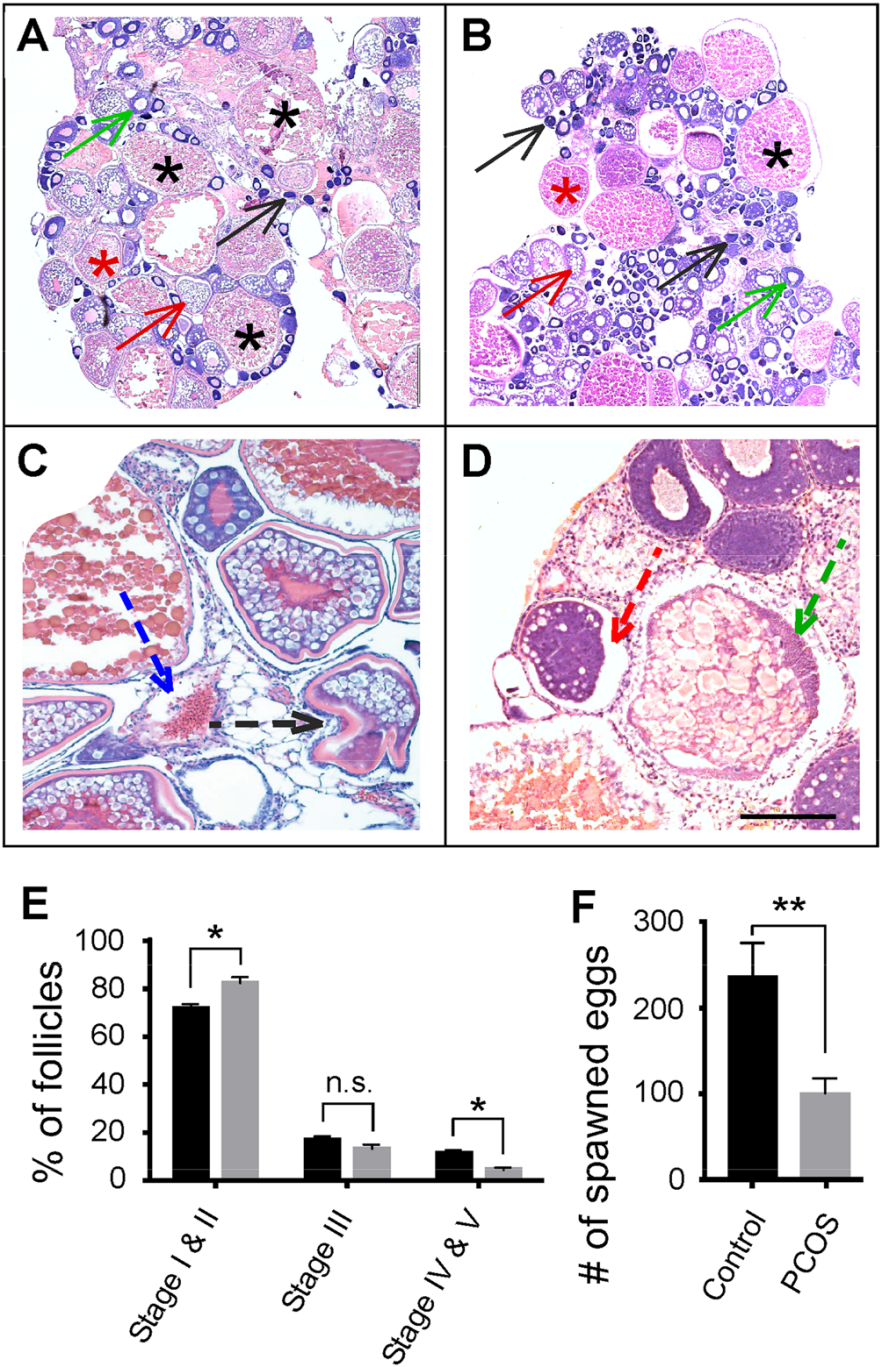
Follicle development and ovulation in control and PCOS zebrafish. (**A**,**B**) Histological sections of the ovary. In control fish (**A**), follicles at different stages could be identified. These include developing follicles (Stages I and II, black and green arrows), transitioning follicles (Stage III, red arrows), and mature follicles (Stages IV and V, red and black asterisks). In PCOS fish (**B**), more developing follicles but less mature follicles were observed. (**C**,**D**) Histological sections that show abnormal follicles in PCOS fish. These include zona pellucida invagination (black arrow), theca hypertrophy/granulosa cell invagination (blue arrow), basal membrane disintegration (red arrow), and basophilic granular accumulation (green arrow). Scale bar: 100 µm in panels (**A**) and (**B**), and 50 µm in panels (**C**) and (**D**). (**E**) Percentage of developing, transitioning, and mature follicles in control (black bars) and PCOS fish (grey bars). Note the increase of developing follicles and the decrease of mature follicles in PCOS animals. (**F**) Ovulation in control (black bar) and PCOS (grey bar) zebrafish. Note the decrease of ovulation in PCOS fish. Data represents the means ± SE (n = 8 in each group). *p < 0.05; **p < 0.01; *ns* not significant.

In control zebrafish, developing (Stage I and Stage II), transitioning (Stage III), and mature (Stages IV and V) follicles can be readily identified (Fig. 2A). In PCOS zebrafish, due to follicular growth-arrest, more follicles remained at developing stages as compared to control animals. While the number of transitioning follicles was similar between the PCOS and control animals, the amount of mature follicles in PCOS fish was significantly reduced (Fig. 2B). In addition, in PCOS fish, atretic follicles were observed, accompanied by other types of defective follicles, such as deformation of zona radiate membranes, detachment of zona radiate from granular cells, invasion of hypertrophy and hyperplasia granulose cells, and accumulation of basophilia materials (Fig. 2C,D). In control fish, the percentages of developing, transitioning, and mature follicles accounted for 72.1%, 16.6%, and 11.3% of total follicles, whereas in PCOS fish, they accounted for 82.3%, 13.4%, and 4.3% of total follicles (Fig. 2E). Due to the decrease in the number of mature follicles, ovulation in PCOS fish was also dropped by 57.7% compared to control animals (Fig. 2F).

In PCOS fish, the ovaries were enlarged. This was revealed by the increase of the gonadosomatic index (GSI). In control fish, the GSI was 0.14 ± 0.01, whereas in PCOS fish, the GSI increased to 0.16 ± 0.01 (p < 0.05; Fig. S2A).

### Abnormal gene expressions in PCOS zebrafish

**Figure 3:**
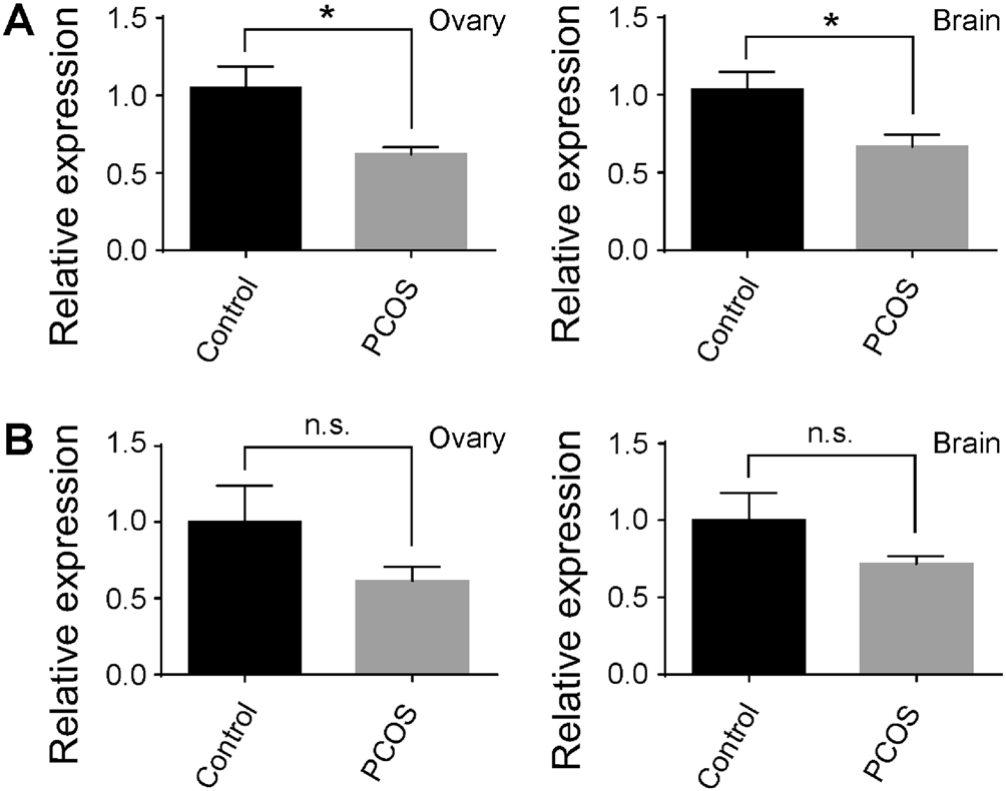
The expression of *Tox3* (**A**) and *Dennd1a* (**B**) in isolated ovary and brain tissues from the control (black bars) and PCOS (grey bars) animals. The expression of *Tox3* and *Dennd1a* in the control sample was normalized to 1, and their expression in PCOS fish was compared to the base value of 1. Note the decrease of *Tox3* expression in PCOS samples. However, no significant changes in *Dennd1a* expression were detected. Data represents the means ± SE (n = 8 in each group). *p < 0.05; *ns* not significant.

To verify the applicability of testosterone-induced zebrafish as a model for PCOS, we examined the expression of two PCOS susceptible genes, *Tox3* and *Dennd1a*, in the control and PCOS animals. In isolated ovary and brain tissues, the expression of *Tox3* was decreased by 43.1% and 36.5%, respectively, in PCOS fish in comparison to its expression in control samples (p < 0.05; Fig. 3A). The expression of *Dennd1a* was also decreased in PCOS fish as compared to its expression in control samples (by 38.9% and 28.3%), but the decrease in either organ was not statistically significant (p > 0.05; Fig. 3B).

### POCS zebrafish showed reduced sexuality levels and mating rates

**Figure 4:**
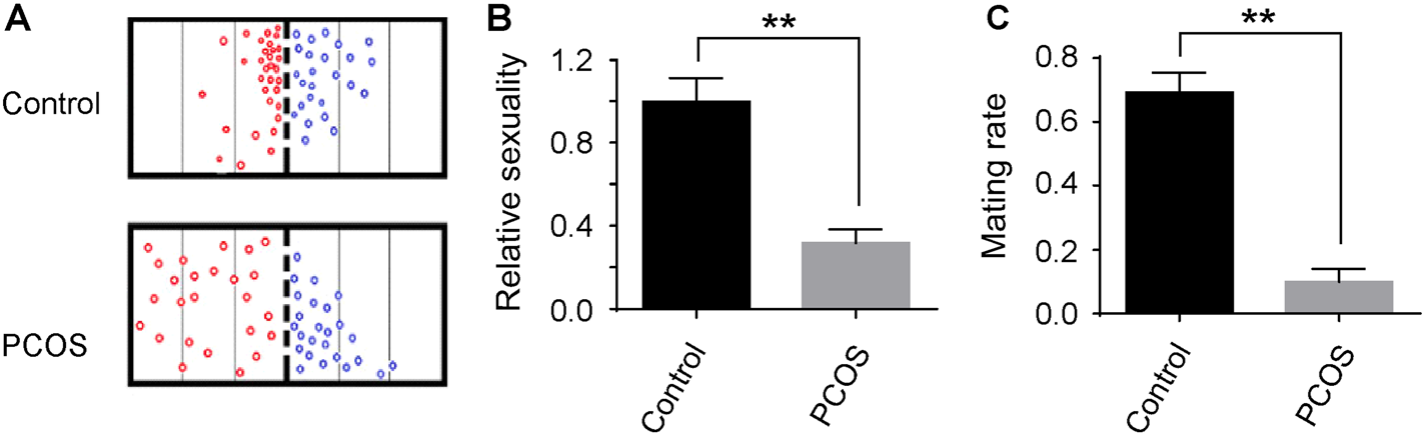
Swimming patterns, sexuality scores, and mating rates of control and PCOS zebrafish. (**A**) Static positions of control (top panel, red circles) and PCOS fish (bottom panel, red circles) at 15-s intervals during a 30-min recording period in the morning. Bold dashed lines indicate the net. In the control setting, the female showed preferences to swim in areas close to the net. In the experimental setting, the PCOS fish swam in all areas, either near or far from the net. Blue circles show the static positions of the male fish at 15-s intervals. (**B**) Sexuality scores of control females (black bar) and PCOS fish (grey bar). The sexuality levels of control fish were normalized to 1, and the sexuality levels of PCOS fish were compared to the base value of 1. Note the decrease in sexuality levels in PCOS fish. (**C**) Mating rates of control (black bar) and PCOS fish (grey bar). Note the decrease of mating in PCOS fish. Data represents the Means ± SE (n = 12 in each group). **p < 0.01.

We examined the sexual and mating behaviors of the control and PCOS fish. In the control experiment (e.g., a control female fish and a control male fish that were placed in the same cage, but separated by a net in the middle of the cage; see Fig. 4A, top panel), for much of the time during the 30-min recording period, both the female and male fish swam in areas close to the net. Often, the fish increased their swimming speed and bumped into the net in an attempt to interact with each other. In the experimental setting (a PCOS female and a control male that were placed in the mating cage, but separated by a net in the middle of the cage; see Fig. 4A, bottom panel), the PCOS fish showed no preference to swim in areas that are close to the net. Instead, for most of the time during the 30-min recording period, the PCOS fish slowly and randomly swam in all areas of its compartment. Based on their swimming behaviors (i.e., affinity to areas that were close to the net, speed of swimming, and number of times the fish bumped to the net), we scored the sexuality levels (i.e., the intention for mating) of the control and PCOS animals. As compared to control fish, the sexuality levels of PCOS fish were decreased by 68.3% (Fig. 4B).

After the behavioral tests, we removed the net in the middle of the cage and allowed the fish to mate for one hour. We calculated the mating rate of PCOS and control fish (percentage of PCOS fish that laid eggs after natural mating) and determined the hatching rate of developing embryos from each mating (e.g., percentage of embryos spawn at 5 days post-fertilization). In PCOS fish, both the mating (Fig. 4C) and hatching rates (Fig. S3A) were decreased as compared to control animals.

### Increase of sexuality levels and reproduction in PCOS fish after rearing in separation from males

**Figure 5:**
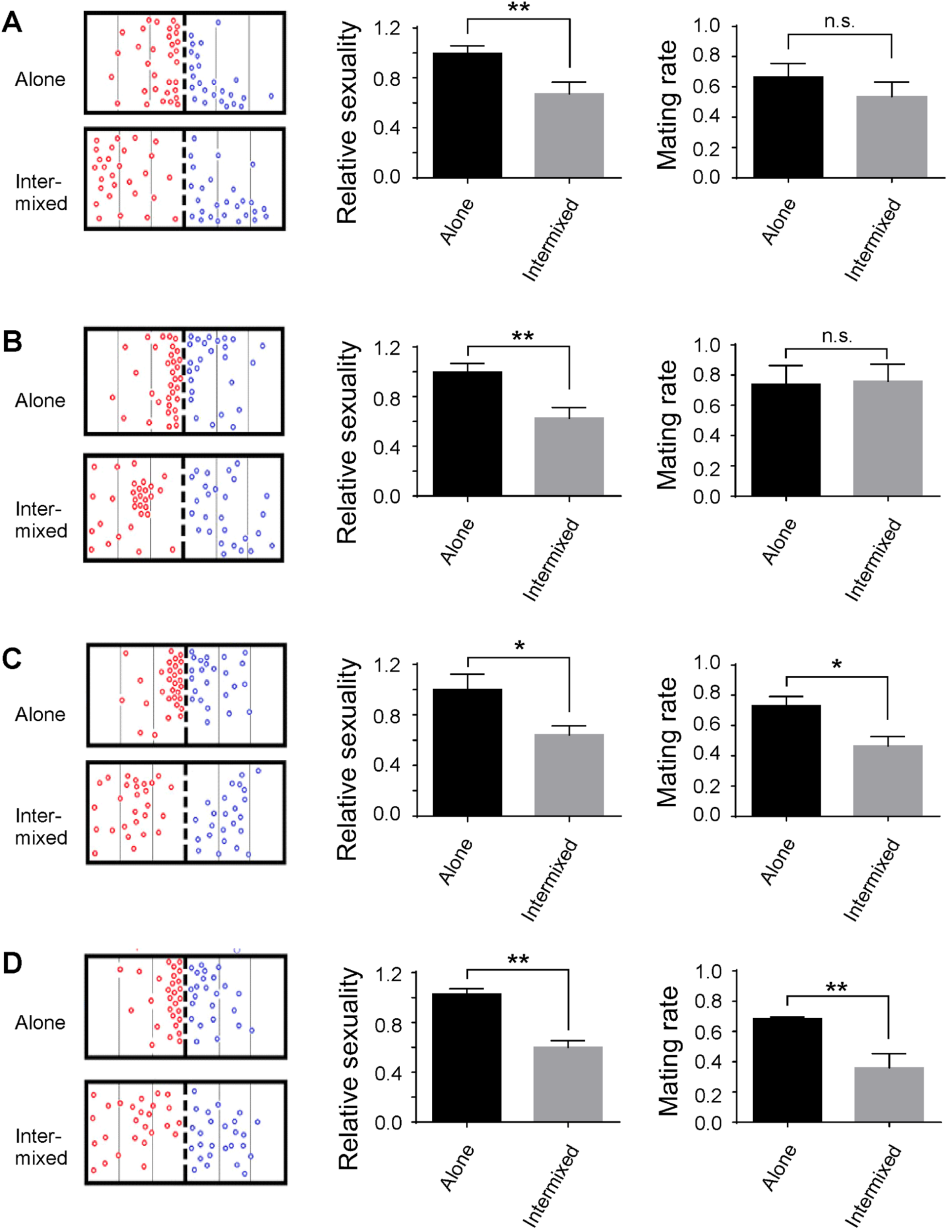
Swimming patterns, sexuality levels, and mating rates of PCOS zebrafish after 3 days (**A**), 14 days (**B**), 30 days (**C**), and 60 days (**D**) of rearing in different conditions. Left panels showed the static positions of the fish at 15-s intervals during the 30-min recording period. Bold dashed lines indicate the net. In all cases, the PCOS fish that had been previously kept alone displayed preferences to swim in areas close to the net. In contrast, the PCOS fish that had been previously kept with males swam randomly in all areas. Middle panels showed relative sexuality scores of PCOS fish that had been previously kept alone (black bars) or intermixed with males (grey bars). The scores of PCOS zebrafish kept alone were normalized to 1, and the scores of PCOS zebrafish that were kept with males were compared to the base value of 1. Note the decrease in sexuality levels in PCOS fish reared intermixed with males. Right panels showed the mating rate of PCOS zebrafish after they were kept alone (black bars) or intermixed with males (grey bars). After 3 or 14 days of rearing under different conditions, no statistical differences were detected in the mating rates. After 30 or 60 days of different rearing, the mating rate was increased in PCOS fish that had been previously reared alone as compared to PCOS fish that had been previously reared intermixed with males. Data represents the means ± SE (n = 8 in each group). *p < 0.05; **p < 0.01; *ns* not significant.

We developed a scheme that kept PCOS fish under different rearing conditions (e.g., either reared alone, or intermixed with males) to test the potential improvement of sexuality and reproduction. After 3 days of rearing alone (one fish per tank, with normal feeding schedules), the PCOS fish showed increased sexuality levels as compared to the PCOS fish that had been previously intermixed with male animals. For example, when they were tested in the mating cage that contained male animals, during the 30-min behavioral test period, the PCOS fish that had been previously reared alone spent more time swimming in areas next to the net in an attempt to mate with the male fish (Fig. 5A; left top panel). In contrast, the PCOS fish that had been previously intermixed with males swam around in all areas in the mating cage, and they showed no preference for swimming next to the net (Fig. 5A; left bottom panel). The change of swimming behaviors was also observed in PCOS fish reared alone for 14, 30, and 60 days (Fig. 5B–D; left top panels). In PCOS fish that were intermixed with males, after 14, 30, and 30 days of rearing, when they were tested for mating behaviors, no obvious changes in swimming patterns were observed; they showed no preference for swimming next to the net in an attempt to mate with the male (Fig. 5B–D; left panels).

Based on their swimming patterns (when tested in the mating cage with males), we calculated the sexuality levels of PCOS zebrafish that had been maintained under different conditions (either alone or intermixed with males). In all cases (e.g., after 3, 14, 30, or 60 days of different rearing), the sexuality levels of PCOS fish that had been reared alone were significantly higher than the sexuality levels of PCOS fish that had been previously reared intermixed with male animals (Fig. 5A–D; middle panels).

To demonstrate if the increase in sexuality of PCOS fish reared alone may correlate with the gradual recovery of ovary functions, we compared the GSI of PCOS fish that had been reared under different conditions. After 3 or 14 days rearing alone or intermixed with males, no significant differences were detected in the GSI in PCOS fish. After 30 or 60 days of differential rearing, the GSI was significantly decreased in PCOS fish that had been kept alone as compared to the GSI in PCOS fish that had been kept intermixed with males (Fig. S2B–E).

To assess whether the fertility levels were changed in PCOS fish that had been maintained under different conditions, PCOS fish were allowed to mate with males for 1 h. From each pair-wised sexual mating, we counted the number of PCOS fish that laid eggs, the number of eggs from each mating, and the number of eggs that were spawned at 5 days post-fertilization. After 3 or 14 days of different rearing, we observed no significant differences in the mating rate between the PCOS zebrafish that had been previously reared alone or intermixed with males (Fig. 5A,B; right panels). When tested after 30 or 60 days of rearing under different conditions, the mating rate was increased in PCOS fish that had been previously reared alone as compared to the mating rate in PCOS fish that had been previously reared intermixed with males (Fig. 5C,D; right panels). In all cases, however, the hatching rate remained unchanged regardless of whether the PCOS fish had been previously kept alone or intermixed with males (Fig. S3B–E).

## Discussion

Testosterone has been known as one of the most influential factors in regulating sexual behaviors in males^1,42–46^. The relationship between testosterone and female sexual activity has also been examined, but the conclusion is ambiguous^47,48^. Some research (e.g., in monkeys) suggests that testosterone is not associated with sexual motivation in females^49^. Other research indicates that androgens (including testosterone) are required for female sex activity. For example, in adrenal-ectomized female monkeys, in which the production of testosterone was decreased, their sexual receptivity and mating rate were decreased^50^. While the underlying mechanisms remain to be further studied, it is possible that testosterone plays an important role in the modulation of sexual hormone circulation in the brain in females.

In humans, there is no direct evidence that correlates the total testosterone levels and female sexual activities. Some studies suggested that high testosterone levels (e.g., in PCOS patients) do not affect the sexual desires in women^51^. In other studies, it is found that testosterone increases the sexual desire in women by enhancing the function of female-specific hormones, such as estrogen^52^. Our data suggests that, based on the behavioral studies using PCOS zebrafish models, higher levels of testosterone reduce the sexual activity in females. For example, after treatment with a high concentration of testosterone (e.g., 100 ng/ml), when encountering male animals, the PCOS fish showed no desire to initiate or engage in sexual copulation. We did not detect histological (follicle distribution and maturation in the ovary) or behavioral (sexual mating) changes in female zebrafish that were treated with lower concentrations of testosterone (e.g., 0.25, 2, and 10 ng/ml).

While the total number of the follicles in PCOS and control fish were similar, in PCOS fish, the majority of the follicles remained in Stages I and II, while the number of mature follicles (e.g., those in Stages IV and V) were decreased. This may be due to the malfunction of endocrine signal transduction in the ovary, by which the process of follicle maturation is arrested. The increase of GSI in PCOS fish supports the notion that high levels of testosterone cause molecular and cellular abnormalities in the ovary. We showed that high levels of testosterone interrupt the patterns of gene expression associated with PCOS (Fig. 3). Previous studies have shown that both genes are involved in cell development and proliferation^10–12^. The decrease of *Tox3* expression may be attributed to the arrest of follicle development and maturation in testosterone treated animals.

Due to the aforementioned molecular, cellular, and tissue abnormalities, the PCOS fish show defects in sexual activity and reproduction (Fig. 4). Under certain circumstances (i.e., by rearing the PCOS fish alone instead of mixing them with males), however, the sexuality levels of PCOS fish may be increased. While the underlying mechanisms remain to be further studied, it is possible that the increase in sexuality is due to the recovery of ovary functions. For example, after 30 days of rearing alone, the GSI was reduced (Fig. S2), the testosterone level was dropped (Fig. S4A), and the number of atretic follicles was decreased (Fig. S4B). We did not detect differences in the hatching rate between the PCOS fish that had been reared alone or intermixed with males (Fig. S3), suggesting that alterations in sexual behaviors may not impact the condition of mature follicles.

It was noticed that in the mating experiments involved with PCOS females, the male zebrafish became less-interested in engaging in sexual behaviors. For example, during the 30-min recording period, the male fish spent less time swimming in areas next to the net as compared to the male fish in the control setting (see Fig. 5). This may be caused by alterations in testosterone levels in the male fish when it encountered a female fish with higher levels of testosterone. Previous studies have shown that in men, the testosterone levels may be varied in accordance with the ovulating state of women. That is, males who were exposed to scents of ovulating females recorded higher testosterone levels than males who were exposed to scents of non-ovulating females^53^. It is possible that, in our experiments, after being exposed to a female (e.g., the PCOS fish) with decreased ovulating cues, the testosterone levels in male fish were decreased, which in turn, decreased their motivation to engage in sexual mating.

In summary, we demonstrate that zebrafish is an excellent model for studying reproductive diseases, such as PCOS. Previously, zebrafish have been used for research in human reproductive systems. Most of those studies emphasized for understanding the molecular and cellular mechanisms involved in the etiology and/or pathology of the diseases, such as defects in gene expression^54–59^, signal transduction^60–64^, cell metabolism^65–69^, or hormone regulation^70–74^. Our study suggests that zebrafish can also be a model system for behavioral studies of human reproductive system diseases. The newly developed schemes (e.g., rearing the PCOS fish alone rather than mixed with males) may provide the proof-of-concept for developing new strategies for treatment of PCOS.

## Supplementary Information

Supplementary Information.
