# Supplementary material for: Improving the sexual activity and reproduction of female zebrafish with high testosterone levels
Source: Sci Rep. 2021 Feb 15;11:3822. doi: 10.1038/s41598-021-83085-4 (PMC7884839; doi:10.1038/s41598-021-83085-4)
Supplement: Supplementary file 1 — Supplementary Information. [file 41598_2021_83085_MOESM1_ESM.docx]

**Supplementary Information**

**Improving the sexual activity and reproduction of female zebrafish with high testosterone levels**

Congcong Liu ^1, 2^, Sophie Yue ^2^, Joshua Solarz ^2^, Jessica Lee ^2^, Lei Li ^2^

^1^ Center for Reproductive Medicine, Shandong University, Jinan 250012, China

^2^ Department of Biological Sciences, University of Notre Dame, Notre Dame, IN 46556, USA

**Supplementary Figures**

**Fig. S1.** A diagram that shows the sequence of major experiments in this research. Adult female zebrafish were treated with different concentrations of testosterone (dissolved in swimming water), then the fish were tested for testosterone levels in the ovaries and the brain. Animals that were treated with 100 ng/ml testosterone (referred to as the PCOS fish) and the control fish were also examined by molecular, cellular, and behavioral assays. In addition, the control and PCOS fish were tested after being reared in different conditions (either alone or intermixed with males) for 3, 14, 30, and 60 days.


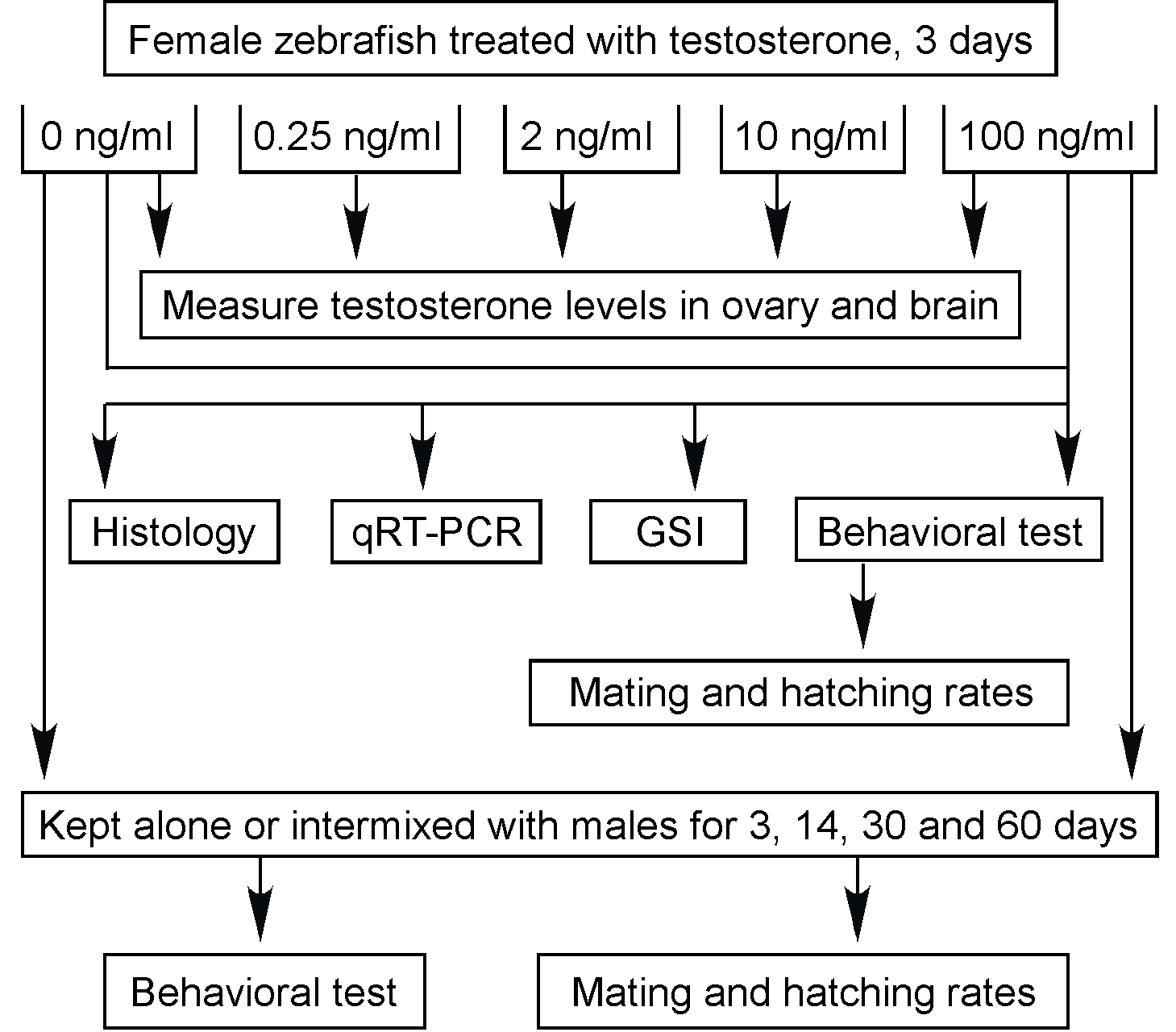


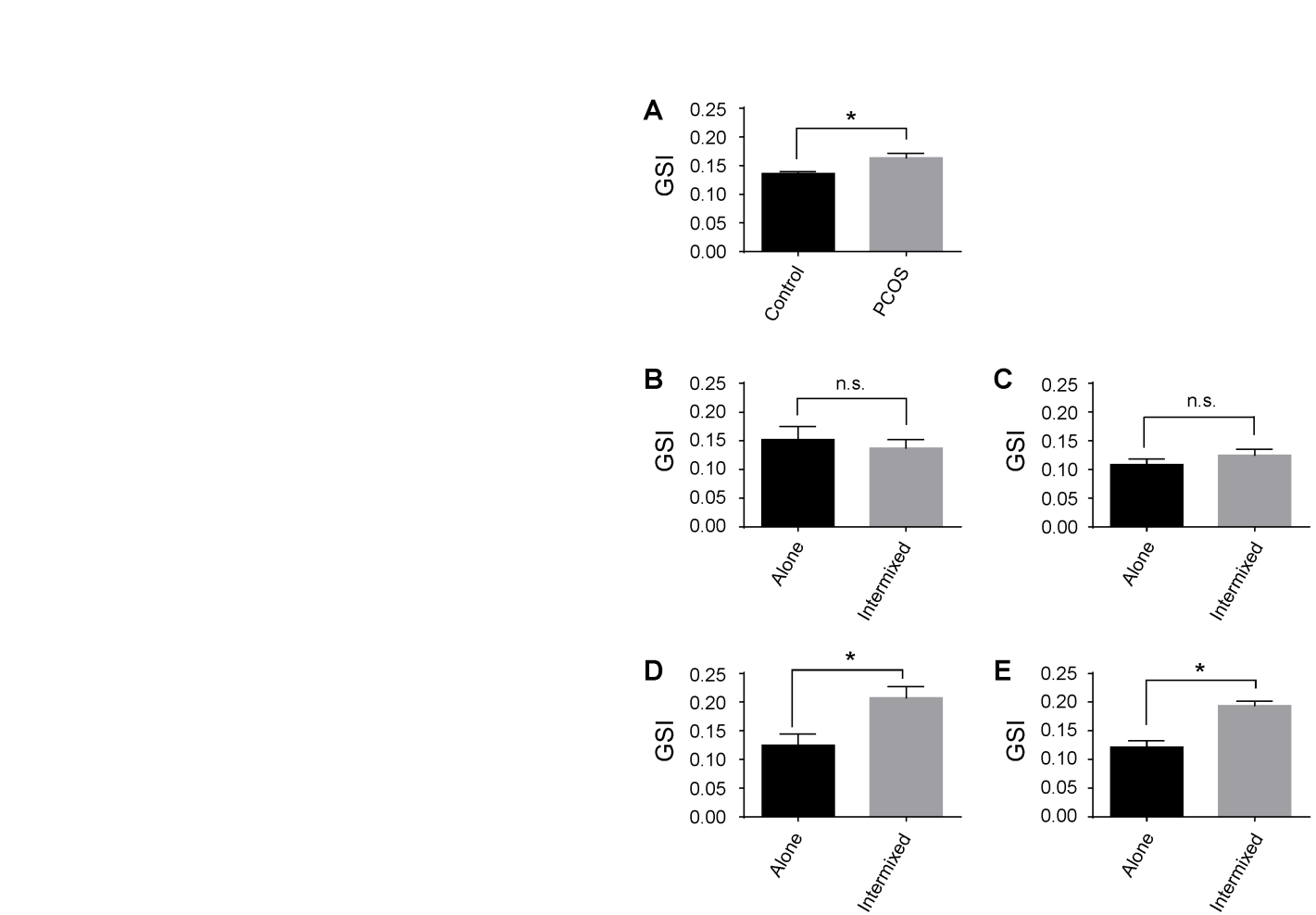


**Fig. S2.** (A) The GSI of control (black bar) and PCOS zebrafish (grey bar). Note the increase of GSI in PCOS fish. (B-E) The GSI of POCS fish measured after 3, 14, 30, and 60 days of rearing alone (black bars) or intermixed with males (grey bars). After 3 or 14 days of different rearing (B, C), no differences in the GSI were detected between the PCOS fish reared alone or previously intermixed with males. After 30 or 60 days of different rearing (D, E), the GSI was decreased in PCOS fish reared alone. Data represents the Means ± SE (n=8-12 in each group). * p < 0.05; ** p < 0.01; n.s., not significant.

**Fig. S3.** (A) Hatching rates of control (black bar) and PCOS zebrafish (grey bar). Note the decrease of hatching in POCS fish. (B-E) Hatching rates of POCS zebrafish after 3, 14, 30, and 60 days of rearing alone (black bars) or intermixed with males (grey bars). No statistical differences were detected regardless of whether the fish were previously kept alone or intermixed with males. Approximately 2900 eggs were collected after mating (2000 from the control group and 900 from the experimental group), and the hatching rate was determined at 5 days post-fertilization. Data represents the Means ± SE (n=8-12 in each group). * p < 0.05; n.s., not significant.


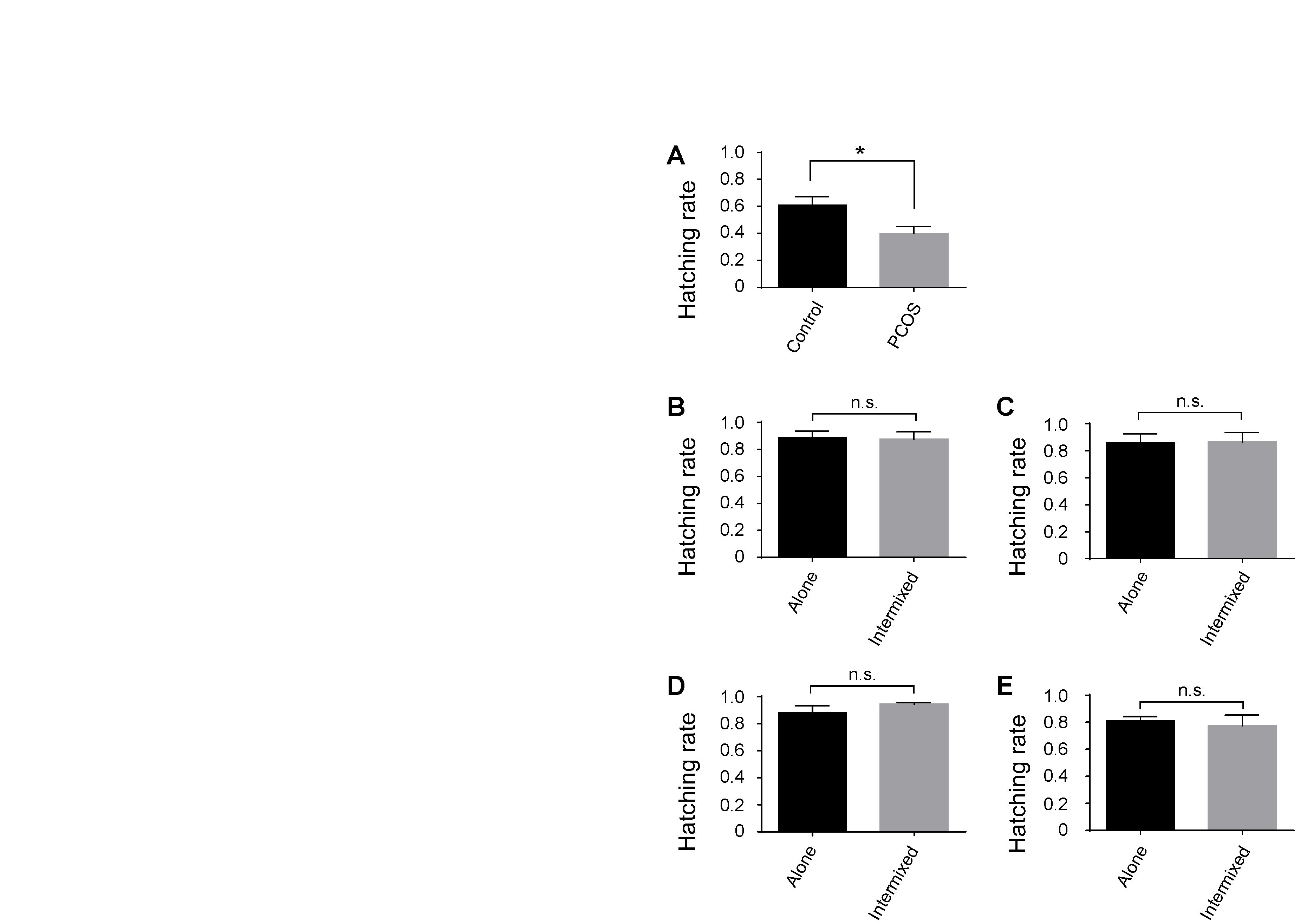

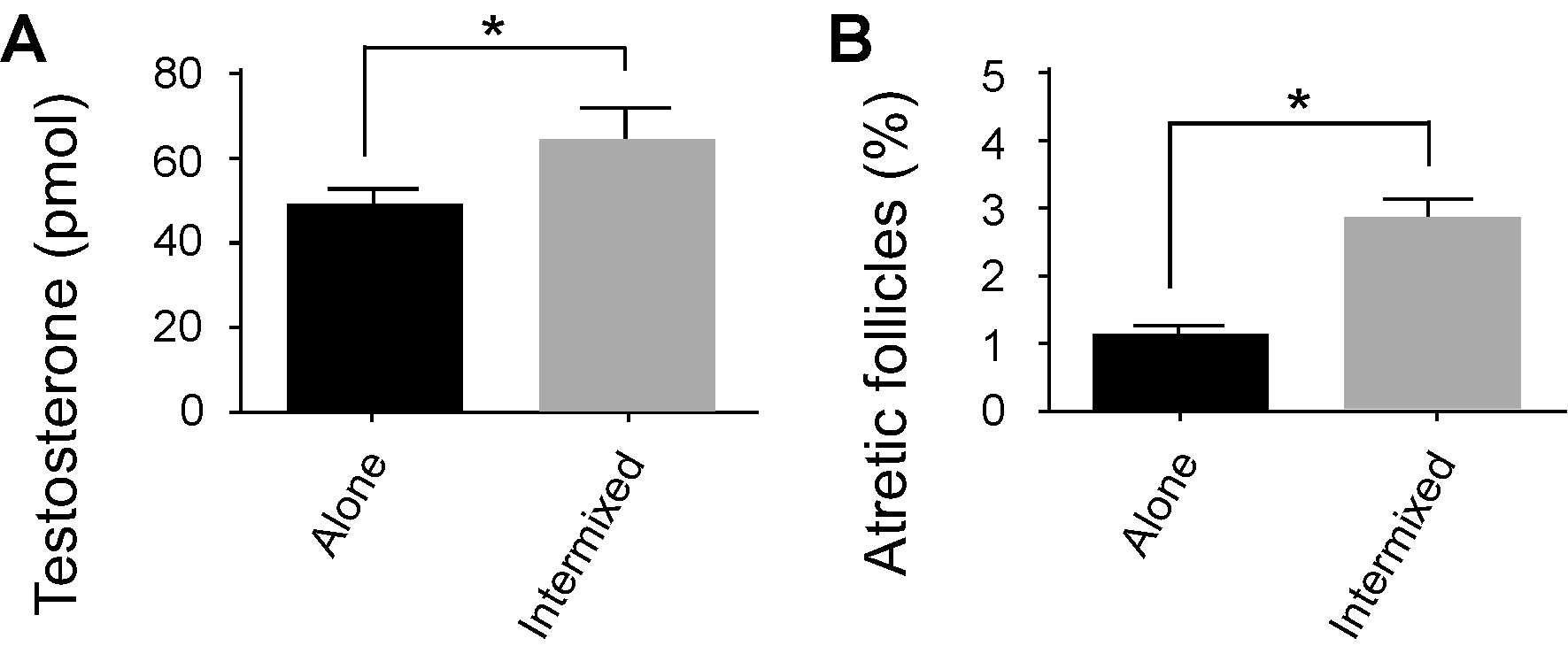


**Fig. S4.** (A) Testosterone levels in isolated ovaries in PCOS zebrafish after 30 days of rearing alone (black bars) or intermixed with males (grey bars). Note the decreases in testosterone levels in PCOS zebrafish reared alone. (B). Numbers of atretic follicles in PCOS zebrafish after 30 days of rearing alone (black bars) or

intermixed with males (grey bars). Note the decreases of atretic follicles in PCOS zebrafish reared alone. Data represents the Means ± SE (n=8 in each group). * p < 0.05.
